# Geometric constraints of endothelial cell migration on electrospun fibres

**DOI:** 10.1038/s41598-018-24667-7

**Published:** 2018-04-23

**Authors:** Maqsood Ahmed, Tiago Ramos, Paul Wieringa, Clemens van Blitterswijk, Jan de Boer, Lorenzo Moroni

**Affiliations:** 1 University of Twente, Department of Tissue Regeneration, Enschede, 7500 AE The Netherlands; 2 Faculty of Engineering, University of Oporto, 4200-465 Porto, Portugal; 3 Maastricht University, Department of Complex Tissue Regeneration, Maastricht, 6200 MD The Netherlands; 4 Maastricht University, Cell Biology Inspired Tissue Engineering, Maastricht, 6200 MD The Netherlands

## Abstract

Biomaterial scaffolds that can form a template for tissue growth and repair forms the basis of many tissue engineering paradigms. Cell migration and colonisation is an important, and often overlooked, first step. In this study, fibrous guidance structures were produced via electrospinning and the effect of physical features such as fibre diameter (ranging from 500 nm to 10 μm) on endothelial cell migration was assessed. Using a modified wound healing assay, fibre diameter was found to have a significant effect on the rate of wound closure and the peak migration velocity of the cells with scaffold diameter shown to influence both morphology and alignment of the migrating cells. The expression, phosphorylation and distribution of focal adhesion kinase (FAK) was disrupted on the different scaffolds with small-diameter scaffolds exhibiting increased FAK phosphorylation with the kinase present in the cytosol whereas on large-diameter scaffolds FAK was largely restricted to focal adhesions at the cell periphery. This study demonstrates that electrospun scaffolds can be used to model cell migration on fibrous substrates, and particularly for the studying effects of physical features of the substrate, and that FAK is a key mediator of cell-scaffold interactions on migrating cells.

## Introduction

The collective movement of cells is fundamental in a number of biological processes in development and disease. Aberrant migration can have profound consequences and has been implicated in pathologies as diverse as intellectual disability and cancer metastasis^1,2^. The mechanisms underlying migration are complex and have yet to be fully elucidated. Cell intrinsic factors such as cellular polarity and adhesion are critical determinants in coordinating the movement of cells, and this core machinery can be further modulated by the extracellular matrix (ECM) to elicit different modes of migration in a context dependent manner^3,4^.

Cells encounter a broad range of extracellular environments including a diverse set of ECM proteins with distinct biochemical properties capable of binding to specific cell receptors that can provoke a range of migratory phenotypes. Meanwhile, matrix stiffness and deformability is highly heterogeneous and can vary by several orders of magnitude across tissue. Cells are able to sense and respond to these mechanical cues through actomyosin cables resulting in tension across the cell, which if asymmetric can lead to cell movement^5^. Finally, the ECM provides a substrate for cells to move across and in this way, matrix geometry and topography are vital parameters in regulating migration^6^. ECM can limit the lateral spreading of the cell – termed confinement – resulting in reduced adhesion to the substrate and increased migration velocities^7^. Moreover, the substrate can induce contact-guided migration across a continuous surface such as a basement membrane, or alternatively a discontinuous surface consisting of free space which can impede migration by restricting the available cell-substrate contact area and thus limiting the degree of traction force the cell can generate^3,6^. Whilst these multiple intrinsic and extrinsic factors can all mediate cell migration individually, it is likely that they act interdependently in a synergistic or antagonistic manner necessitating a more holistic approach to understanding how cells sense and respond to their environment during migration.

Cell migration is of particular importance in the field of tissue engineering and regenerative medicine where biological scaffolds are often deployed as templates to guide tissue repair in organs damaged by injury or disease. The success of this paradigm is dependent on the successful integration of the scaffold to the host tissue and vasculature, which can then supply the scaffold with the necessary nutrients and oxygen to promote repair. Migration of endogenous endothelial cells (ECs) from pre-existing vessels in the neighbouring tissue is an important first step. Whilst factors such as adhesion molecules and growth factors have been shown to play a central role in facilitating migration into the scaffold, how the physical properties of the scaffold mediate this process is less well understood. Features such as pore size and porosity have been shown to have a role in scaffold vascularization with large, interconnected pores shown to promote blood vessel ingrowth^8–10^. A more complete understanding of scaffold properties which can create a permissive environment for endothelial cell migration and angiogenesis is imperative to facilitate the improved design criteria for the next generation of tissue scaffolds.

Electrospinning is a facile technique capable of producing fibrous scaffolds that mimic the morphology of native ECM. Fibre diameters can range from a tens of nanometres to a several micrometres. This study presents a quantitative analysis of the influence of fibre diameter of electrospun scaffolds on the migration of human umbilical vein endothelial cells (HUVECs) using a physical barrier assay. By exploiting high-content imaging, cell morphologies were evaluated and the differential expression of known migratory markers was examined at the gene and protein level.

## Materials and Methods

### Scaffold fabrication and characterisation

Scaffolds were electrospun according to the parameters outlined in Table 1. Briefly, poly(lactic-co-glycolic acid) (PLGA, Corbion Purac Biomaterials, Netherlands) was dissolved in 1,1,1,3,3,3-Hexafluoro-2-propanol (HFiP, BioSolve BV) and electrospun using standard electrospinning apparatus in an environmentally controlled (25 °C, 30% humidity) chamber. A parallel plate collector system with a 14 mm diameter untreated coverslip was used to obtain aligned fibres. Parameters were adjusted (Table 1) to obtain fibres with a range of diameters.

**Table 1:** Electrospinning parameters used for scaffold fabrication (HFiP: hexafluorosiopropanol, DCM: dichloromethane).

Scaffold	Polymer concentration (%w/v)	Solvent	Flow rate (ml/h)	Time (min)	Voltage (kV)
S0.5	25	HFiP	1.5	12	17.5
S1	40	HFiP	1.5	6	17.5
S2	75	HFiP	1	4	17.5
S4	75	HFiP	1.5	5	17.5
S10	75	HFiP/DCM (1:1)	4	12	17.5

Fibre morphology and diameter were characterised using scanning electron microscopy (SEM, Philips XL 30 ESEM-FEM) after gold sputter-coating. Fibre diameter was calculated from 12 images per sample using an automated Fiji (NIH, Bethesda, MD) macro developed in-house.

### Cell culture

**Figure 1:**
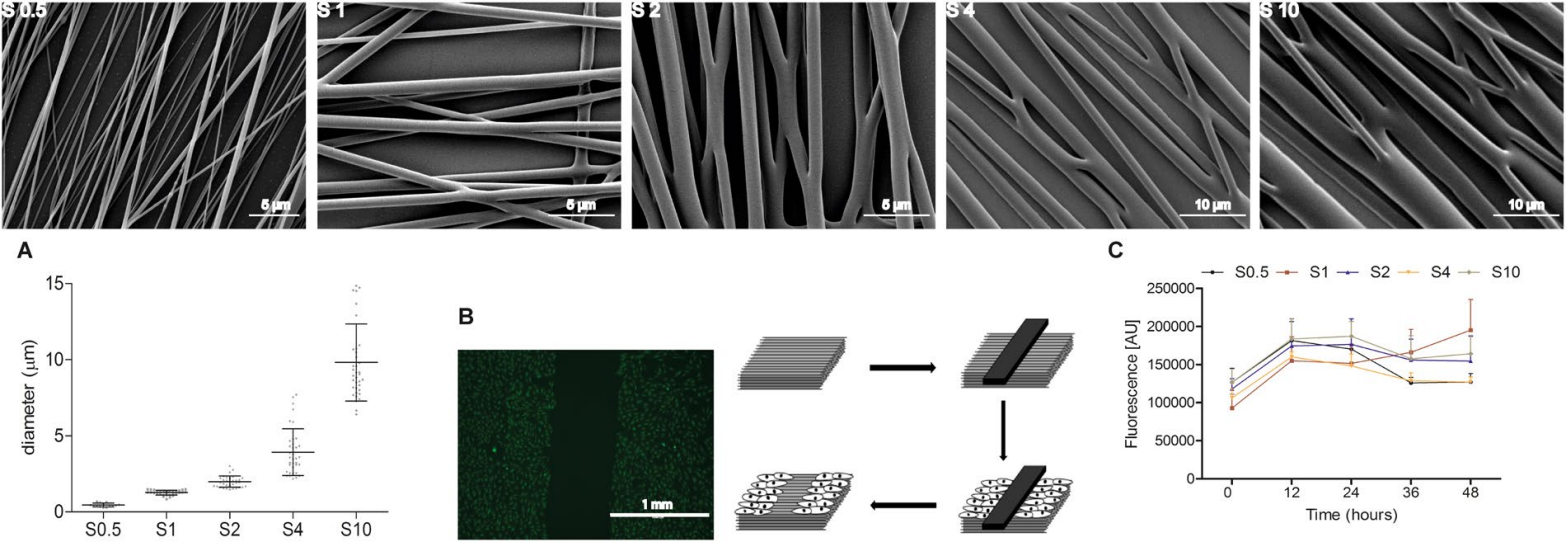
SEM images of different diameter electrospun scaffolds used for measuring cell migration. (**A**) Quantification of fibre diameter on each scaffold (mean ± standard deviation). (**B**) Experimental set-up of modified wound healing assay using a physical barrier placed perpendicular to the direction of the aligned fibres to create open space consisting of fibres aligned towards the direction of migration for cells to move in to. (**C**) Cell proliferation over a 48 hour period demonstrating no significant difference between the scaffolds.

Primary HUVECs (Lonza, passage 4–8) were cultured in endothelial growth medium (EGM, cat. Number: CC-3162), which consisted of endothelial basic medium supplemented with (%V/V): 2% foetal bovine serum (FBS), 0.04% hydrocortisone, 0.4% human fibroblasts growth factor B (hFGFB), 0.1% VEGF, 0.1% R3-Insulin-like Growth Factor-I (R3-IGF-1), 0.1% ascorbic acid, 0.1% human endothelial growth factor (hEGF), 0.1% Gentamicin/Amphotericin-B (GA-1000), and 0.1% heparin (all from Lonza). Cells were grown at 37 °C in a humid atmosphere with 5% CO_2_. The media was refreshed every other day. Previously sterilized scaffolds (70% ethanol for 2 hours) were incubated in EGM medium overnight. After media removal, the physical barrier (1 mm by 13 mm) was placed in the centre of the scaffold perpendicular to the aligned electrospun fibres. In this way, a central gap of aligned fibres for the cells to migrate into is created (as depicted in Fig. 1) and held using rubber O-rings (Eriks, The Netherlands). Cells were then seeded at 2 × 10^4^ cells/cm^2^ and returned to the incubator. Upon reaching confluence, the physical barrier was physically removed using sterile tweezers and the samples washed thrice with phosphate buffered saline solution (PBS, cat. Number: 10010-023, Life Technologies). Fresh medium was added and the downstream experiments conducted.

### Viability assay

Cell viability was assessed using PrestoBlue^®^ assay according to the manufacturer’s protocol (Life Technologies, cat. Number: A-13262). Briefly, 10% (V/V) of PrestoBlue^®^ reagent was added in each well (n = 3) and incubated at 37 °C for 2 hours. Three 100 µL media samples were transferred from each well into a Nunc™ 96-well plate (Thermo Scientific, cat. Number: 267350). Fluorescence was measured at 540–570 nm excitation 580–610 nm emission in VICTOR^3^™ 1420 Multilabel Counter (PerkinElmer). The readout from the scaffolds was corrected with a blank (medium plus PrestoBlue^®^ reagent).

### Migration assay

HUVECs were treated with 10 µM Cell Tracker™ Green CMFDA (Molecular Probes®, cat. Number: C7025) for 45 minutes at 37 °C. At the start of each assay the physical barrier was physically removed using sterile tweezers and subsequent images of each sample were taken (EVOS^®^ XL microscope, Life Technologies) in triplicates at 0, 2, 12, 24, 36, and 48 hours using x10 lens. The images were then analysed using a Cell Profiler (Broad Institute) pipeline, developed by the authors and described in detail previously^11^.

### Cell morphology assessment

In a bid to identify morphological features of the migratory cells on the different diameter scaffolds, cells were fixed at the experimental midpoint (24 hours) by removing the cell culture media, PBS wash (3X), fixation with 4% (w/v) paraformaldehyde for 10 minutes and a further PBS wash (3X) at room temperature (RT). The samples were blocked using 1% (v/v) foetal bovine serum for 1 hour and permeabilised using 1% (v/v) triton x-100 for 15 minutes. They were then stained with Alexafluor 594 phalloidin (Life Technologies, 1:100 dilution) for 1 hour and DAPI (Invitrogen, 0.1 mg/ml) for 15 minutes before visualisation under a fluorescent microscope (Nikon Eclipse E600).

The shape descriptors were calculated from at least 20 images from 3 independent experiments per condition, using a semi-automated script from ImageJ. Briefly, a binary threshold method was applied to the images followed by the identification of cells using a ROI tool. The shape descriptors (FeretX, FeretY, Feret angle and circularity) were then calculated and analysed using Matlab R2013a (Mathworks) and plotted in a Rose diagram.

### Biochemical analysis

#### Gene expression

Total RNA was isolated using a combination of the TRIzol^®^ method with the NucleoSpin^®^ RNA II isolation kit (Bioké, cat. Number 740955.50). Briefly, samples were washed once with PBS and 500 µl of TRIzol® reagent (Invitrogen) was added to each well containing the samples. The TRIzol solution was pipetted up and down several times to ensure complete lysis and collected for RNA isolation. Chloroform (200 µL) was added to the TRIzol solution and centrifuged (15 mins, 12000 *g*, 4 °C), resulting in the colourless, aqueous phase containing the RNA being collected whilst the solvent and scaffold debris being discarded. The aqueous solution was mixed with 250 µL of 70% ethanol and loaded onto the RNA binding column of the kit. Subsequent steps were done in accordance with the manufacturer’s protocol. RNA was collected in RNase-free water and its quantity and quality analysed using an ND100 spectrophotometer (Nanodrop technologies). 200 ng of RNA was used for first strand cDNA synthesis using iScript (Bio-Rad). 1 μL of undiluted cDNA was used for subsequent analysis. PCR was performed in an iQ5 real time PCR machine (Bio-Rad) using SYBR Green supermix (Bio-Rad) and fold increase calculated following ΔΔCt method comparing gene expression in migrating cells with that of non-migrating HUVECs using GAPDH as a reference gene. Primer sequences are shown in Table 2.

**Table 2:** List of primer sequences used for qPCR.

Genes	Forward	Reverse
GAPDH	GGATTTGGTCGTATTGGG	GGAAGATGGTGATGGGATT
Profilin	TCAAGTTTTTACGTGAATGGGCT	CGAAGATCCATGCTAAATTCCCC
FAK	GCTTACCTTGACCCCAACTTG	ACGTTCCATACCAGTACCCAG
VCL	CCAAGATGATTGACGAGAGACAG	AGAGGTGAGTTGTAACACACGA
VEGF	AGGGCAGAATCATCACGAAGT	AGGGTCTCGATTGGATGGCA
FGF	TGCGTCGTGGAGAACAAGTTT	GCACGGTAACGTAGGGTGTG
IL8	TTTTGCCAAGGAGTGCTAAAGA	AACCCTCTGCACCCAGTTTTC

#### Protein quantification

Commercially available direct enzyme linked immunosorbent assays (ELISA, Cat No: KHO0431 and KHO0441, Life Technologies) were used to determine the total and phosphorylated levels of focal adhesion kinase (FAK). Cells were lysed on the scaffolds using the recommended cell extraction buffer. Lysates were collected and stored at −80 °C and the standard manufacturers protocol was followed thereafter. FAK and pFAK protein expression was normalised to that in resting HUVECs.

#### Antibody staining

cells cultured on scaffolds were fixed using paraformaldehyde 4% (W/V), pH 7.0–7.2 for 15 minutes and blocked in PBT: 0.1% (V/V) Triton X-100 supplemented with 1% (W/V) BSA (cat. Number A9418) in PBS pH 7.4 for 1 hour at RT, all from Sigma-Aldrich. Rabbit anti-FAK ([pS732] polyclonal antibody, Life Technologies™) was added at concentration 1:500 in PBT and kept overnight at 4 °C. Goat anti-rabbit 594 (1:1000 in PBT, Life Technologies™) was added for 1 hour at RT. The samples were rinsed twice with PBT and followed by an additional wash with PBS for 10 minutes at RT. For nuclei staining DAPI (1:1000 in PBS, Sigma-Aldrich) was used and incubated for 15 minutes at RT. The sample coverslips were then mounted onto microscope slides using one drop of aqua polymount (around 20 µL) and images acquired (Nikon E600).

### Statistical analysis

A one-way analysis of variance (ANOVA) followed by Tukey’s multiple comparison test (unless otherwise specified) was used to determine statistically significant differences (GraphPad Prism). Data are expressed as mean ± standard deviation (unless otherwise specified).

## Results

### Scaffold characterisation

Fibrous scaffolds with a diameter ranging from 500 nm to 10 μm were successfully produced using a parallel-plate electrospinning set-up resulting in aligned fibres (Fig. 1A). Diameter was adjusted by modifying the electrospinning parameters summarised in Table 1. In this way, scaffolds with a defined diameter could be produced reproducibly. Five scaffolds were produced: S0.5, S1, S2, S4 and S10 corresponding to scaffolds with an approximate diameter of 0.5, 1, 2, 4 and 10 μm, respectively. Fibre diameters are normally distributed with the mean and standard deviation of each scaffold detailed in Fig. 1A, namely 474 ± 23 nm (S0.5), 1.26 ± 0.16 μm (S1), 1.98 ± 0.37 μm (S2), 3.91 ± 0.53 μm (S4) and 9.81 ± 1.53 μm (S10).

### Cell migration

A modified scratch wound assay was developed to measure the collective migration of an endothelial monolayer. A physical barrier produced from Teflon was placed in the centre of a 24 well plate to create free space for the cells to migrate into; thus, despite the name, no scratch or wound injury took place. Cells were seeded and allowed to reach confluence around the Teflon barrier. At confluence, the barrier was removed creating a well-defined linear border over lengths of the order of a few millimetres (Fig. 1B). In this way, a reliable and reproducible model system was created with minimal cell damage.

HUVECs were seeded onto the scaffolds and adhered to the electrospun fibres (Fig. 1B). As the pore size is relatively minimal compared to the size of the cells and the underlying coverslip was untreated for cell culture, we did not observe adhesions forming on the substrate. Viability was assessed using a PrestoBlue assay over a 48-hour time period and no significant differences were detected between the scaffolds, suggesting metabolic activity does not vary across the scaffolds and thus it is unlikely that there is a significant difference in proliferation on the scaffolds (Fig. 1C, p = 0.4914, n = 3, two-way ANOVA).

Upon removal of the physical barrier, the cells at the leading edge became motile and began to progressively occupy the denuded area consisting of aligned fibres in the direction of migration. The cells were tracked for 48 hours, capturing three images per scaffold at 0, 2, 12, 24, 36 and 48 hours; allowing the simultaneous analyses of cell movements on the five different scaffolds. Using an automated image analysis pipeline described previously, the effect of the underlying fibre diameter on the overall displacement of the leading edge and cell velocity was examined.

**Figure 2:**
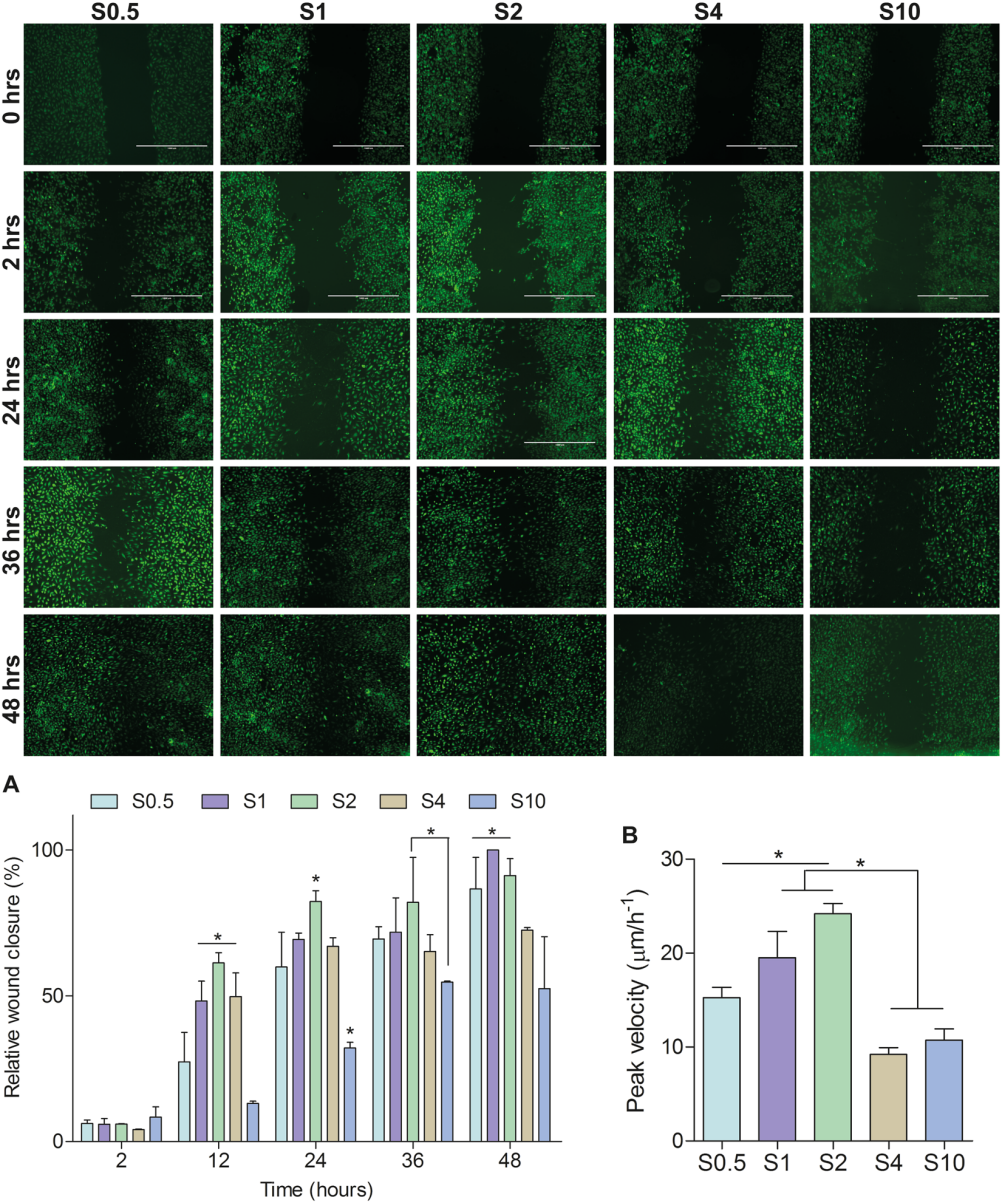
Fluorescent images of migrating cells on each scaffold over a 48 hour time period. (**A**) Quantification of relative wound closure as described previously^11^. (**B**) Peak migration velocity, found 12 hours after removal of physical barrier. Mean ± standard deviation, n = 3, *p < 0.05, ANOVA.

The covered area represents the displacement of the cell sheet. After 2 hours, no significant differences can be detected between the scaffolds (Fig. 2A). However, after 12 hours substrates with an intermediate fibre diameter (S1, S2 and S4) elicit the greatest cell displacement with the relative wound closure significantly higher than S0.5 or S10. At 24 and 36 hours, migration of HUVECs on S10 is still impaired whereas there is now no significant difference between the cells on S0.5 and those on S1 and S4. S2 displays a slight statistically significant increase in relative wound closure. After 48 hours, there is no significant difference between S0.5, S1 and S2 with the denuded area close to 100% covered, however the wound has failed to close on S4 and S10 suggesting diminished migratory phenotype.

Examining the migration velocities of the cells on the different substrates revealed a peak velocity at approximately 12 hours, which then progressively decreased. On the 1 and 2 μm diameter scaffolds the velocity at 12 hours reached a peak of 19.52 ± 3.91 μmh^−1^ and 24.18 ± 1.55 μmh^−1^ respectively (Fig. 2B). The remaining scaffolds had significantly reduced peak velocities: S0.5: 15.26 ± 1.92 μmh^−1^ S4: 9.22 ± 0.52 μmh^−1^, S10 10.73 ± 2.01 μmh^−1^ (n = 3, p < 0.0001, ANOVA).

### Cell morphology

**Figure 3:**
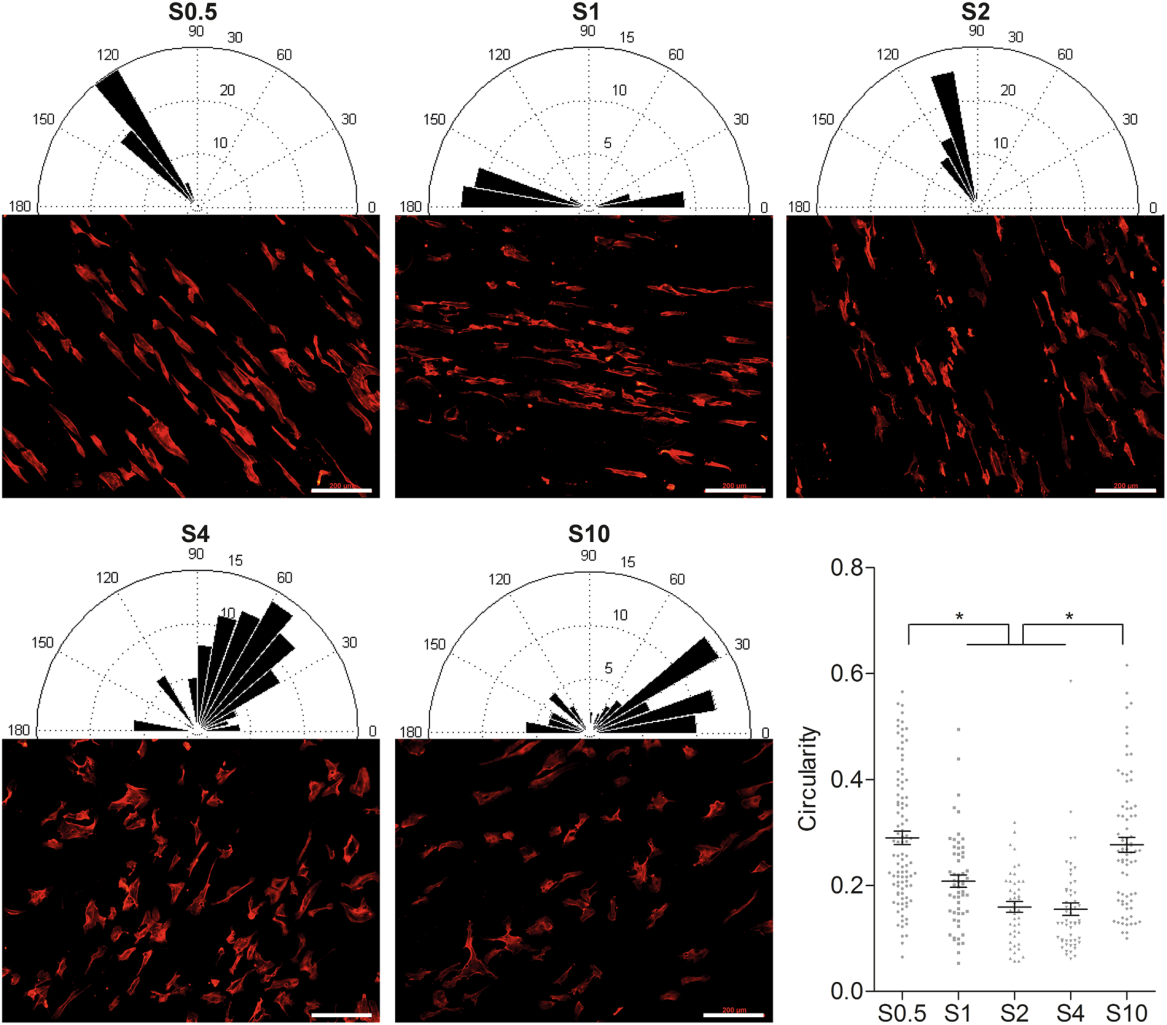
Cell morphology and alignment on scaffolds. Fluorescent images of cells cultured on each scaffold with corresponding Rose plot of HUVEC alignment suggesting cells on larger diameter scaffolds are more randomly orientated whereas cells on S0.5, S1 and S2 are relatively uniformly orientated and aligned in one direction. Cell morphology is quantified in terms of circularity where cells on intermediate scaffolds (S1, S2 and S4) exhibit a significantly reduced circularity and are therefore more elongated (scale bar: 200 μm, mean ± standard deviation, n = 3, *p < 0.05, ANOVA).

We next examined the cell alignment and morphology of the individual cells on the different fibre diameter scaffolds (Fig. 3). Cells on S0.5, S1 and S2 appear more uniformly aligned in one direction, with the cells occupying just one or two 10° bins in the Rose diagrams (Fig. 3). Meanwhile, cells on S4 and S10 occupy multiple bins suggesting a much greater range of orientations.

We next scrutinised the extent of cellular polarisation by measuring the cell circularity, a ratio of the cell short axis over its long axis with a value close to 0 indicating an elongated cell whilst a value of 1 suggests a perfectly spherical cell. The intermediate scaffolds (S1, S2 and S4) contained significantly (ANOVA, p < 0.05, n = 3) more elongated cells with a lower circularity measurement (0.20, 0.16 and 0.16 respectively) compared to S0.5 and S10 (0.29 and 0.28).

The morphological descriptors suggest that the cells on S0.5 are highly aligned in one direction, although they are not as polarised as those on S1 and S2 where the cells are significantly more polarised and aligned in a uniform direction. Meanwhile, S4 exhibits polarised cells but not in a uniform direction whereas S10 contains cells that are neither aligned or particularly polarised.

### Biochemical analysis

We next investigated any biochemical changes that may have occurred in the migrating cells on the different scaffolds. Quantitative PCR was used to determine transcriptional differences in well-established pro-migratory growth factors and adhesion proteins which were then further validated at the protein level through ELISA’s and immunofluorescence.

**Figure 4:**
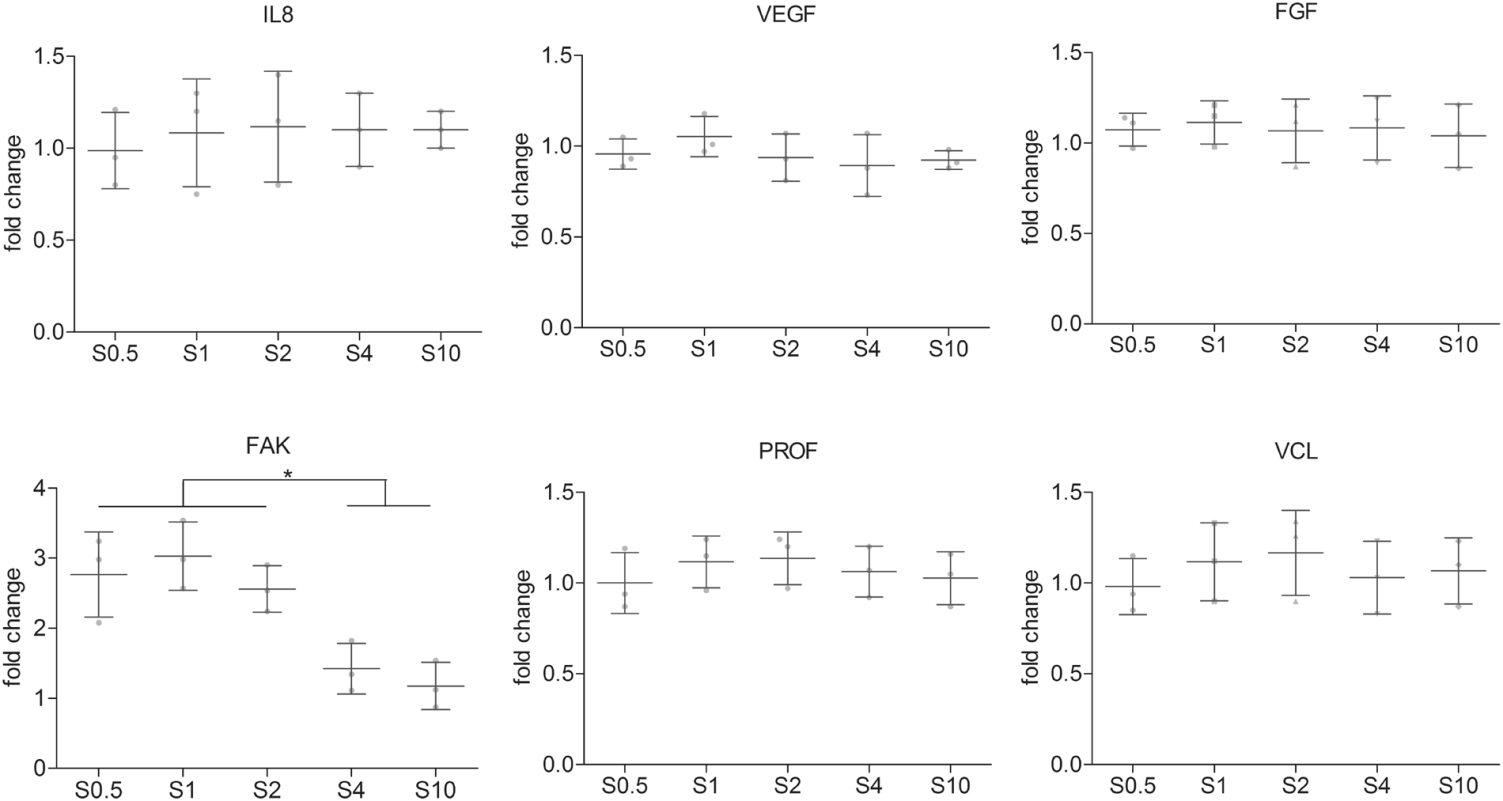
Gene expression profiles of HUVECs on the different scaffolds. No significant differences detected between the genes except FAK which was upregulated on small diameter scaffolds compared to S4 and S10 (mean ± standard deviation, n = 3, *p < 0.05, ANOVA).

Cell lysates from the different scaffolds were collected after 12 hours corresponding to peak migration velocity. Differential expression pattern of a panel of 6 genes was assessed (Fig. 4). No significant differences between the scaffolds were detected in the expression of three soluble pro-migratory cues (IL8, VEGF and FGF). Focal adhesion kinase (FAK) expression was found to be significantly increased on the three smaller scaffolds (S0.5, S1 and S2) compared to S4 and S10 (ANOVA, N = 3, P = 0.0012). No significant differences were detected in the expression of vinculin and profilin on the scaffolds.

**Figure 5:**
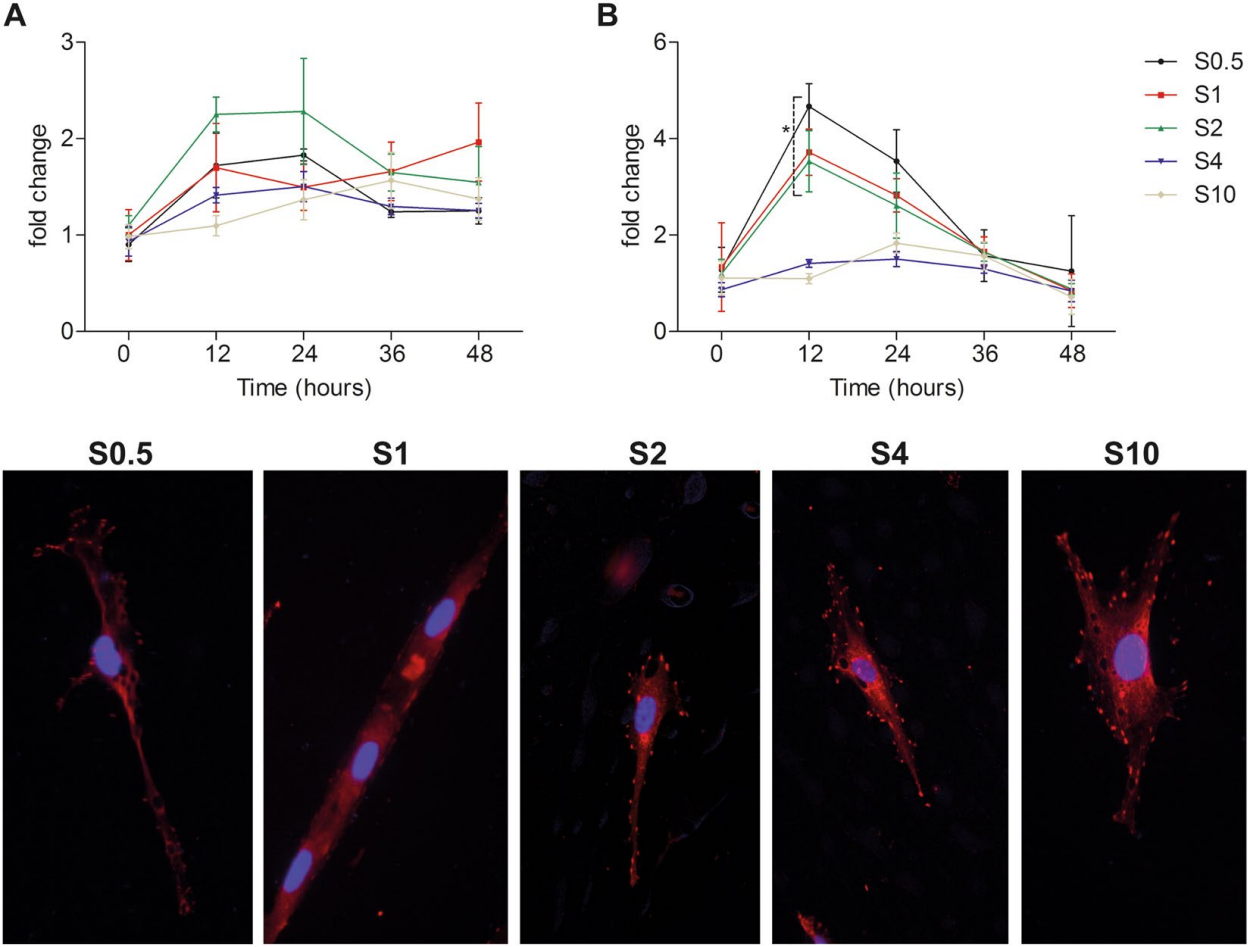
FAK protein expression. (**A**) Total FAK ELISA over 48 hours indicating that no significant difference in total FAK protein levels. (**B**) However, FAK phosphorylation was significantly higher at 12 hours on the small diameter scaffolds (mean ± standard deviation, n = 3, *p < 0.05, ANOVA). Phospho-FAK immunofluorescence images display pFAK distribution over the cells cultured on the different scaffolds showing a greater degree of pFAK localisation to focal adhesions on the cell periphery on large diameter scaffolds whereas on the small diameter scaffolds, pFAK distribution is restricted to the cytosol.

Total FAK levels were determined using ELISA and whilst levels were highest on S2, no significant differences were detected (Fig. 5A). However, when measuring the extent of FAK phosphorylation on the different scaffolds, there was a significant increase (2-way ANOVA, p < 0.001, n = 3) in phospho-FAK at 12 hours on S0.5, S1 and S2.

We next determined the cellular distribution of FAK through immunofluorescence (Fig. 5). On the more polarised cells on S0.5, S1 and S2 to a degree, FAK appears to be largely cytosolic. Indeed, on the S0.5 and S1, few if any focal adhesions can be detected. Meanwhile, S4 and S10 have significant number of focal adhesions positive for FAK.

## Discussion

Electropsun scaffolds are beginning to find numerous applications as ECM analogues for the regeneration of a range of different tissues due to similar structural and morphological features as the native ECM. The central premise of this regenerative approach is the migration and colonisation of the scaffold by endogenous cells from the surrounding tissue. In this study, we investigated the directional migration potential of a HUVEC monolayer on these synthetic scaffolds and assessed how fibre diameter influences the speed and extent of migration using a modified scratch wound assay. Others have presented different approaches to promote directional cell migration using growth factors^12,13^, electric fields^14^, stiffness gradients^15^ and swelling gradients^16^.

Scaffolds were generated using a parallel-plate electrospinning technique with subtle changes to the processing conditions yielding aligned fibres with diameters ranging from 0.5–10 μm. The air gap created between the two parallel electrodes used for scaffold fabrication generates residual electrostatic repulsion resulting in alignment of the charged fibres^17^. The diameter of the electrospun fibres was dependent on variables such as polymer concentration, solvents used and flow rate. Large diameter scaffolds were generated in co-solvents with higher concentrations of polymer and faster flow rates. An increased mass of extruded polymer either due to a more concentrated solutions or faster extrusion rate combined with a rapidly evaporating solvent would result in fibres with large diameters (S10) due to reduced time for whipping and thinning out of the fibre.

High-content imaging and a semi-automated image analysis platform allowed the analysis of migration rates across the scaffolds. A stable cell tracker signal that did not decay over time was obtained using 10 μM of CMFDA cell tracker. HUVECs migrated in a diameter dependent manner on the scaffolds that was independent of cell proliferation and therefore wound closure was primarily due to cell migration into the denuded area rather than cell proliferation.

Cells on a surface devoid of any physical constraints tend to form multiple lateral pseudopodia resulting in random migration. Whereas upon seeding HUVECs to electrospun scaffolds consisting of aligned fibres, migration was largely unidirectional exhibiting a biphasic relationship with migration potential at its peak on S2 with significantly reduced migration rates on smaller and larger diameter scaffolds, particularly the 4 and 10 μm fibre diameter scaffolds. The differential was particularly pronounced when examining the initial migration velocities immediately after withdrawing the physical barrier. HUVECs on the intermediate scaffolds (S1 and S2) displayed significantly increased peak migration velocities after 12 hours that began to gradually taper off as the denuded gap was being filled. The varying migration velocities could be the result of cellular morphology and alignment of the HUVECs on the different scaffolds with the intermediate scaffolds provoking a highly aligned, polarised population of cells ready to migrate into the exposed area. To examine this hypothesis, images were collected and analysed for both cell alignment and morphology. Indeed, the cells were found to be significantly more aligned and polarised on S1 and S2 suggesting the contact guidance and polarisation elicited by the underlying fibre diameters could play a role in dictating migration. Similar findings have been reported previously demonstrating a strong correlation between the degree of cell elongation and the distance that endothelial cells migrate upon endothelium injury^18,19^. CDC42, a member of the Rho GTPase family, has been suggested as a master regulator of cell polarisation mediated directional migration^13,20^. Whilst soluble signals such as growth factors and chemoattractants are well-established pro-migratory agents able to promote migration over large distances through the activation of internal signalling cascades, local physical cues such as ECM topography can dictate migration rate and directionality through direct interaction in a contact-dependent manner^21–24^. Taken together, the higher degree of alignment observed in cells orientated in a single direction suggested that by inhibiting the lateral spreading of the cells and promoting a uniaxial morphology, S1 and S2 are able to stimulate migration rates. These observations agree with previously published reports on topographical features promoting cell migration^7,25^.

Next, we aimed to identify the biochemical effectors that drive these observations. The expression of 6 genes, 3 well established growth factors/cytokines and 3 adhesion based proteins, was investigated in HUVECs from the different scaffolds at a time point corresponding to peak migration velocity (12 hours). From the transcriptional analysis, no appreciable differences were detected on the expression levels of three pro-migratory factors (IL-8, VEGF and FGF) and neither on the expression of vinculin and profiling, proteins whose functions include the strengthening of cell anchoring to the ECM (commonly associated with a decrease in cell migration) and actin polymerisation, respectively^26,27^. On the other hand, FAK was identified as differentially expressed on S0.5, S1 and S2, confirmed at the protein level via ELISA. FAK is a non-receptor tyrosine kinase associated with focal adhesions and is a crucial regulator of cell shape, adhesion and motility^28–30^. FAK tyrosine kinase activity is involved in the regulation of focal adhesion turnover^31,32^. Taken together, these observations suggest that the major difference triggered by the different fibre diameters are mainly related to focal adhesion complex turnover rather than metabolic changes or formation of more cell-matrix anchorage points. We thereafter examined the temporal expression of FAK over the course of the experiment and whilst no significant difference was found in total FAK expression, a significant increase in phosphorylated FAK was detected at 12 hours corresponding to the peak migration velocity. FAK activity is regulated through post-translational modifications, particularly phosphorylation, indeed phosphorylation of FAK has been shown to be the single critical event in regulating actin and adhesion dynamics^33^. An increase in the phosphorylation of tyrosine 397 phosphoacceptor on FAK has previously been implicated in migrating cells with FAK-deficient cells displaying reduced motility and an increase in the number of focal adhesions^31,32^. The significant higher levels of FAK expression observed in either S0.5, S1 and S2 scaffolds suggests a greater degree of focal adhesion turnover, essential for cell migratory processes^34–36^. In examining the spatial distribution of pFAK across the HUVECs on the different scaffolds, there appeared to be more pFAK at the cell periphery localised to focal adhesions on scaffold S4 and S10, whereas on S0.5, S1 and S2 staining appeared to be more pronounced in the cytosol. The partitioning of FAK between focal adhesions and the cytosol has been suggested as a novel regulatory mechanism of FAK signalling, particularly in regulating focal adhesion disassembly and cell-edge retraction^37^. FAK binding to integrin-cytoskeleton complexes such as talin, filamin, vinculin and tensin forms the basis of a focal adhesion complex^38^. These complexes act as anchorage points for the cells to exert traction and move forward. Subsequently, cells release from the underlying substrate so as to continue cell movement. Thus, directional cell migration requires continuous and coordinated assembly and disassembly of the focal adhesion complexes at both the leading and rear edge of the cell body^39,40^. Therefore, the scattered distribution of the pFAK throughout the cell periphery on S4 and S10 scaffolds may be indicative of a disorganised migratory process resulting in lower velocities and consequently lower coverage of the denuded area. Whereas the formation of the focal adhesion complexes on S1 and S2 preferentially occurred in the longitudinal direction along the fibres, resulting in higher rates of cell elongation and directed cell migration. Phosphorylation of Tyr^397^ was shown to increase in the off-rate of FAK from focal adhesions leading to focal adhesion disassembly and increased cell migration. FAK turnover is faster than other focal adhesion proteins and this rapid flux of FAK may allow the exchange of focal adhesion associated FAK with the cytosolic FAK having modified activity. This may facilitate a greater responsiveness in the cell to migration associated stimuli.

## Conclusions

This study provides evidence to support the notion that cells are highly sensitive to their local microenvironment and can respond to topographical cues to elicit a range of functions. Substrate geometry can induce changes in cell shape and orientation and control migration velocity. These findings suggest that substrate design and scaffold topography are important parameters in determining scaffold efficacy *in vivo* adding an additional layer of complexity to the rational design of tissue scaffolds. Developing facile approaches to manipulate substrate geometry and topography with a degree of spatial control and on a larger scale remains a significant challenge to overcome.
